# Manganese-based Prussian blue nanoparticles inhibit tumor proliferation and migration *via* the MAPK pathway in pancreatic cancer

**DOI:** 10.3389/fchem.2022.1026924

**Published:** 2022-10-24

**Authors:** Shanshi Tong, Zhilong Yu, Fang Yin, Qilin Yang, Juhang Chu, Luyao Huang, Wenxue Gao, Mingping Qian

**Affiliations:** ^1^ Department of General Surgery, Shanghai tenth People’s Hospital, Shanghai, China; ^2^ State Key Laboratory of Oncogenes and Related Genes, Renji Hospital, Shanghai Cancer Institute, Shanghai Jiao Tong University, Shanghai, China; ^3^ Department of Gastroenterological Surgery, Peking University People’s Hospital, Beijing, China; ^4^ Shanghai Engineering Technology Research Center for the Functional Development of Human Intestinal Flora, Shanghai tenth People’s Hospital, Shanghai, China; ^5^ School of Medicine, Tongji University, Shanghai, China; ^6^ Clinical Research Management Office, Shanghai tenth People’s Hospital, Shanghai, China

**Keywords:** MnPB NPs, photothermal therapy, chemodynamic therapy, pancreatic cancer, the MAPK pathway

## Abstract

Pancreatic cancer (PC) is one of the deadliest gastrointestinal malignancies. Advances in molecular biology and surgery have significantly improved survival rates for other tumors in recent decades, but clinical outcomes for PC remained relatively unchanged. Chemodynamic therapy (CDT) and Photothermal therapy (PTT) represent an efficient and relatively safe cancer treatment modality. Here, we synthesized Mn-doped Prussian blue nanoparticles (MnPB NPs) through a simple and mild method, which have a high loading capacity for drugs and excellent CDT/PTT effect. Cell line experiments *in vitro* and animal experiments *in vivo* proved the safety of MnPB NPs. We stimulated the PC cells with MnPB NPs and performed transwell migration assays. The migration of PC cells was reduced company with the decrease of two classical proteins: matrix metalloproteinase-2 (MMP-2) and matrix metalloproteinase-9 (MMP-9). Moreover, MnPB NPs induced ferroptosis, which mediated the MAPK pathway and achieved tumor elimination in nude mice. This effective and safe strategy controlled by irradiation represents a promising strategy for pancreatic cancer.

## Introduction

For a long time, the treatments of pancreatic cancer (PC) were considered limited and difficult. Because PC has an insidious onset, the opportunity for surgery is often lost when frequent clinical presentations appear. As PC incidence increases by 0.5%–1.0% per year, it is expected to become the second-leading cause of cancer-related death by 2030 (Draus et al., 2021). A few advances have been made in disease diagnosis, perioperative management, and surgical skills over the past few decades, but these advances showed limited effects on the overall survival rate (Zhao and Liu, 2020; Neoptolemos et al., 2021). Thus, novel therapeutic strategies are vital for PC treatment (Trunk et al., 2021).

Advances in nanomaterials greatly promote the development of diagnosis and treatment of cancers (Yang et al., 2012). As a novel strategy, nanoparticles make it possible to release drugs accurately, target the tumor microenvironment and kill tumor cells effectively. PTT based on photothermal material is a newly emerging therapy, which could convert light energy into heat for tumor treatment (Chang et al., 2021). Compared with other traditional therapies, there are lots of irreplaceable advantages in PTT (Sun et al., 2021; Zheng et al., 2021; Guan et al., 2022a). Firstly, PTT has no systemic damage and is particularly suitable and effective for superficial solid tumors (Wang W. et al., 2022). Moreover, PTT could be flexibly combined with other imaging examinations to kill cancer cells precisely (Xie et al., 2020). In addition, PTT has many potential advantages such as surprising efficacy in overcoming drug resistance because of the killing of cancer cells. CDT triggers Fenton and Fenton-like reactions induced by endogenous chemical energy, which kill cancer cells by generating hydroxyl radicals (·OH) (Tang et al., 2021; Zhou et al., 2021). The tumor microenvironment (TME) is typically characterized by mildly acidic conditions, defective vascular architecture, overexpression of certain enzymes, high concentrations of glutathione (GSH), and reactive oxygen species (ROS) (Li S. et al., 2020; Wang et al., 2022b). These unique properties of TME allow us to design stimuli-activatable nanoplatforms for cancer therapy. Hence, the concept of CDT has been widely used in the design of anti-tumor nanomaterials (Guan et al., 2022b). Most of these nanomaterials are inorganic transition metals containing redox activity, such as Fe^2+^/Fe^3+^, Cu^+^/Cu^2+^, Mn^2+^/Mn^4+^ and so on. Here, we designed MnPB nanoparticles (MnPB NPs). We assumed that incorporating manganese (Mn) into Prussian blue nanoparticles (PB NPs) could improve therapy efficiency compared to PB nanoparticles alone. Besides, MnPB NPs showed the synergistic effect of PTT and CDT for PC treatment.

Ferroptosis is a novel process based on iron overloaded as well as lipid peroxidation, which has unique morphological and bioenergetic characteristics including mitochondrial shrinkage, increased mitochondrial membrane density, membrane integrity disruption, and NADH depletion (Lei et al., 2022). There has been an explosion of research about ferroptosis and PC in recent years (Kremer et al., 2021; Ye et al., 2021). PC is not responsive to most current treatment options, but targeting the ferroptosis pathway may provide an alternative. Changes in ROS are very critical to the occurrence of ferroptosis (Su et al., 2019). Using ROS generated by CDT to induce ferroptosis for cancer treatment is an attractive option. Moreover, the MAPK signaling pathway is closely related to ferroptosis. Sorafenib, a multikinase inhibitor, an agonist for ferroptosis. However, associations of the MAPK pathway and ferroptosis in nanomedicine still remain unclear.

Herein, we designed and fabricated MnPB NPs to enhance PTT/CDT for tumor therapy. *In vitro* and *in vivo* tests were conducted to determine the anti-tumor efficacy of MnPB NPs. Pancreatic cancer cells were extremely inhibited by MnPB NPs in terms of proliferation and migration. The ferroptosis regulated by the MAPK pathway was activated by MnPB NPs. These presented results indicated that MnPB NPs might contribute to a more effective therapy for pancreatic cancer treatment.

## Materials and methods

### Chemicals and reagents

Manganese chloride (MnCl_2_.4H_2_O), potassium ferrocyanide (K_4_ [Fe (CN)_6_]), and ethanol were purchased from Aladdin Chemistry Co. Lt. We purchased citric acid from Sigma-Aldrich. Milli-Q water was used to produce deionized water (H_2_O).

### Preparation of MnPB NPs

Based on the co-precipitation strategy, we synthesized MnPB NPs by a simple ion-exchange method. First, 0.3 mM of MnCl_2_.4H_2_O and 0.5 mM of citric acid dissolved in 20 ml of deionized water were heated to 60°C for 5 min. As a result, solution A was formed. Solution B was formed by dissolving K_4_ [Fe (CN)_6_] (0.4 mM) and citric acid (0.4 mM) in deionized water (20 ml) and heating to 60°C under magnetic stirring for 5 min. Subsequently, we added solution B dropwise into solvent A and kept it at 60°C for 2 min. The collected product was washed with deionized water and ethanol after cooling to room temperature. In the final step, we dried the washed solution at 60°C for 24 h in a vacuum oven.

### Characterization of MnPB NPs

With an acceleration voltage of 200 kV, transmission electron microscopy (TEM) observations and elemental mapping of nanocomposites were conducted on a JEM-2100 F transmission electron microscope (TEM, JEOL, Tokyo, Japan). Crystallinity and characterization of MnPB NPs was measured using X-ray diffraction (XRD).

### Photothermal effect evaluation

We irradiated MnPB NPs at 100, 200, and 400 μg/ml with an 808 nm near-infrared laser (1.0 W/cm^2^) and recorded the temperatures through an imaging camera (Fotric, Shanghai, China). Besides, we irradiated MnPB NPs solution with a NIR laser, and the solution was naturally cooled to room temperature. We calculated the photothermal conversion efficiency following the published study (Fang et al., 2021).

### Evaluation of the activity to produce ROS

We fully mixed MnPB NPs with methylene blue (MB) solution and H_2_O_2_, and applied UV–vis spectroscopy to record UV–vis spectrum at varied times, which suggested the generation of ROS.

### Cell culture

From the American Type Culture Collection (ATCC), we obtained pancreatic cancer cell line PANC-1 and normal pancreatic cell line hTERT-HPNE, and cultured according to standard ATCC protocols. Briefly, Dulbecco’s Modified Eagle medium (DMEM) containing 10% fetal bovine serum (FBS) was used to culture PANC-1 cells. The hTERT-HPNE cells were cultured as ATCC’s protocol.

### Cytotoxicity assay

Cell viability after treatment was determined using the Cell Counting Kit-8 (CCK-8, Dojindo, Japan) assay. Cells at the density of 1500 per well were seeded in 96-well plates and incubated with MnPB NPs in a 5% CO_2_ incubator for 24 h, then cells were incubated with CCK-8 solution for 90 min. Finally, the absorbance of the solution at 450 nm is measured using a microplate reader (Thermo, United States). A ratio of the absorbance of treated cells to that of control cells was used to calculate cell viability.

### ROS detection assay

In 12-well plates, 3*10^5^ cells were plated per well and treated sequentially with Phosphate Buffered Saline (PBS), NIR, MnPB NPs, and MnPB NPs + H_2_O_2_. Cells were then incubated with 2′,7′-dichlorodihydrofluorescein diacetate (DCFH-DA, 10 μM) in DMEM without FBS for 20 min. The cells were washed three times with PBS, and ROS levels were measured using fluorescence microscopy (Olympus, Tokyo, Japan) afterward.

### Anticancer effect *in vitro*

The Calcine-AM/propidium iodide (PI) test (Beyotime Biotechnology, Shanghai, China) were used following the instructions of manufacturers. We divided the PANC-1 cells into six groups: 1) PBS; 2) NIR; (3)H_2_O_2_; 4) MnPB + NIR; 5) MnPB + H_2_O_2_; 6) MnPB + NIR + H_2_O_2_. After the treatments, we stained the PANC-1 cells with Calcein-AM and PI for 20 min. Images were acquired through fluorescence microscopy (Olympus, Tokyo, Japan).

### Anticancer effect *in vivo*

We suspended pancreatic cancer cells at a density of 1.0*10^7^ cells per ml in cold PBS. Four-week-old BALB/c nude mice were injected subcutaneously with 100 μl of the cell suspension containing 1*10^6^ cells.

Then, the growths of tumors were monitored and measured every 3 days. Measurements of long and short diameters were used to monitor tumor growth, and the following formula was used to calculate tumor volume: V (mm^3^) = width^2^ (mm^2^) × length (mm)/2. We divided the nude mice into four groups: 1) PBS; 2) NIR; 3) MnPB NPs; 4) MnPB NPs + NIR. In order to explore the effect of MnPB NPs, we injected 150 μl PBS into the mice of group 1 and group 2, and 150 μl MnPB NPs were injected into the mice of group 3 and group 4 intravenously. After 8 h, the mice of groups 2 and group 4 were irradiated with a laser (808 nm) for 15 min. To measure the dynamic changes of tumor temperature, a FLIR A300 thermal imaging camera was used. We monitored the tumor volume as well as body weight every 3 days. All nude mice were sacrificed after 18 days, and we conducted immunohistochemistry (Ki67) in tumor tissues, blood routine examination, liver function test, and histological analysis in five major organs.

### Colony formation assay

Initially, 500 cells per well were seeded and cultured in six-well plates. The colonies were stained with 0.1% crystal violet (Sigma Aldrich, United States) after fixation with 4% paraformaldehyde after 2 weeks of culture. A light microscope (Olympus, Tokyo, Japan) was used to assess colonies with more than 50 cells.

### Transwell migration assay

Approximately 3*10^4^ cells were suspended in 200 μl of serum-free medium and seeded in an upper chamber without matrigel. About 800 μl medium with 10% FBS were added to the lower chamber in a 24-well plate. Lower chamber cells were washed, fixed, stained, and photographed after 36 h.

### Western blotting

The cells were lysed in Radio Immunoprecipitation Assay (RIPA) Lysis buffer supplemented with proteases (Yeason, China) and phosphatase inhibitors (Yeason, China) for 20-min incubation on ice. The bicinchoninic acid assay kit (Yeason, China) was conducted to measure the protein concentration in cell lysates. About 8%–12% SDS-polyacrylamide gels were used to separate total protein lysates from different samples, then Polyvinylidene fluoride (PVDF) membranes were electrophoretically transferred (130 V, 50 min at 4°C). The blocking solution was applied to PVDF membranes followed by incubation with specific antibodies overnight at 4°C. The following antibodies were used in this study: anti-β-actin (1:4000, ab8227, Abcam), anti-MMP9 (1:1000, CST, Massachusetts, United States), anti-MMP2 (1:1000, CST, Massachusetts, United States), anti-MEK (1:1000, CST, Massachusetts, United States), anti-p-MEK (1:1000, CST, Massachusetts, United States), anti-p-ERK (1:1000, CST, Massachusetts, United States), anti-ERK (1:1000, CST, Massachusetts, United States), anti-GPX4 (1:1000, CST, Massachusetts, United States).

### Immunohistochemistry

Paraformaldehyde-fixed mouse subcutaneous tumor tissues were washed three times with PBS, which were then transferred into ethanol. Paraffin was used for embedding tissues. Then tissues were dewaxed and stained. The primary antibody was anti-Ki67 (1:2000, CST, Massachusetts, United States).

### Statistical analysis

The statistical analysis was conducted *via* SPSS 22.0 statistical software and GraphPad Prism 5 (Graphpad software). There is a mean and standard deviation (SD) for all data provided. To determine the significance of the difference, Student’s t-test or one-way analysis of variance (ANOVA) was used. *p* < 0.05 was considered statistically significant. *p < 0.05; **p < 0.01; ***p < 0.001; ****p < 0.0001; ns: no significance.

## Results

### Construction of characterization of the MnPB NPs

**FIGURE 1:**
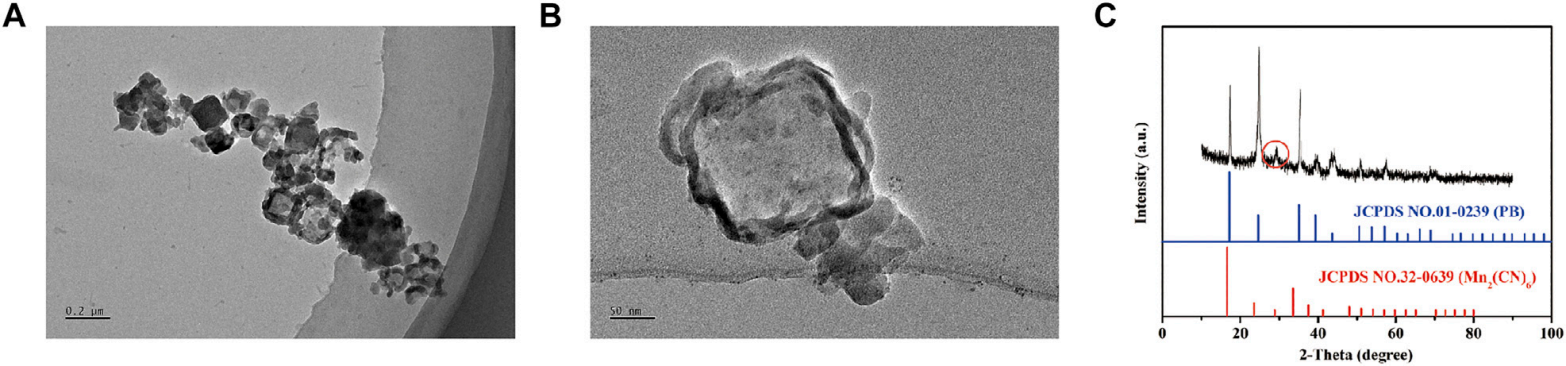
The characteristics of MnPB NPs **(A)** Low- and **(B)** Medium-magnification TEM image of MnPB NPs. **(C)** XRD spectra of MnPB NPs.

The sizes and morphologies of MnPB were analyzed by TEM. The mean diameter of MnPB NPs was 237 nm (Figures 1A,B). In addition, XRD patterns showed the characteristic peaks (Figure 1C) of MnPB NPs (JCPDS No.01–0239, JCPDS No.32–0639), which suggested that MnPB NPs had been synthesized and showed the oxidation state of Mn (+2).

### Photothermal property and generation of ROS

**FIGURE 2:**
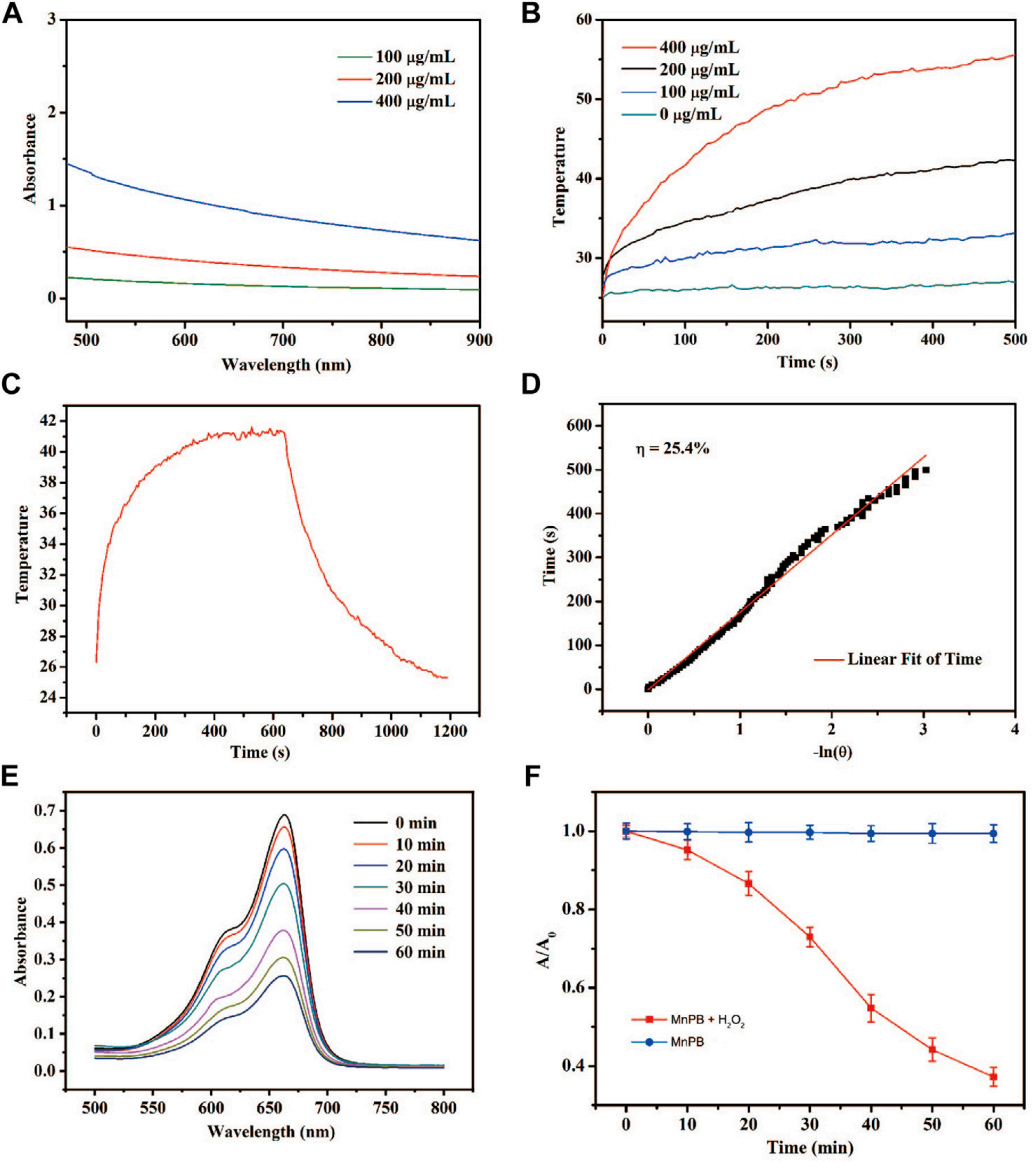
The photothermal properties of the MnPB NPs **(A)** UV-Vis-NIR absorption spectra of MnPB NPs. **(B)** Temperature profiles of MnPB NPs at different concentrations under laser irradiation **(C)** The temperature changes of MnPB NPs (irradiation heating and natural cooling) at 200 μg/ml concentrations **(D)** linear regression for the naturing cooling process. **(E)** Degradation of MB with the treatment of MnPB NPs and H_2_O_2_. **(F)** Absorption of MB in two groups at 664 nm.

As expected, MnPB NPs exhibited broad absorption in aqueous dispersion through the NIR region (Figure 2A), which indicates MnPB NPs might be a potentially good PTT agent. Under NIR light irradiation, MnPB NPs with different concentrations were evaluated for their photothermal properties (Figure 2B). The MnPB NPs at 200 μg/ml rapidly rose to 41°C after irradiation for 10 min (Figure 2C), and the temperature could kill tumor cells irreversibly. Moreover, MnPB NPs were investigated for their photothermal conversion efficiency, which represented the ability to convert light energy into heat energy. The photothermal conversion efficiency of MnPB NPs was up to ∼25.4% (Figure 2D), which was higher than other photothermal agents, such as Cu_2−x_Se (22% only) (Hessel et al., 2011). Thus, these data indicated that MnPB NPs were a promising PPT agent. The TME of pancreatic cancer is rich in H_2_O_2_ and GSH. MnPB NPs would promote the production of ROS only after entering TME. In acidic tumor microenvironment, H_2_O_2_ and Mn^2+^ further generate OH, which could enhance effect of CDT. As showed in Figure 2E, the co-catalytic capacity of MnPB NPs was measured by MB. The characteristic absorption peak intensity at 652 nm decreased significantly over time. In addition, the absorbance of MB in presence of MnPB NPs decreased much faster than the control group (Figure 2F). These data suggested that MnPB NPs are expected to enhance CDT in the TME of pancreatic cancer.

### The MnPB NPs induce intracellular ROS and cell death in PC cells

**FIGURE 3:**
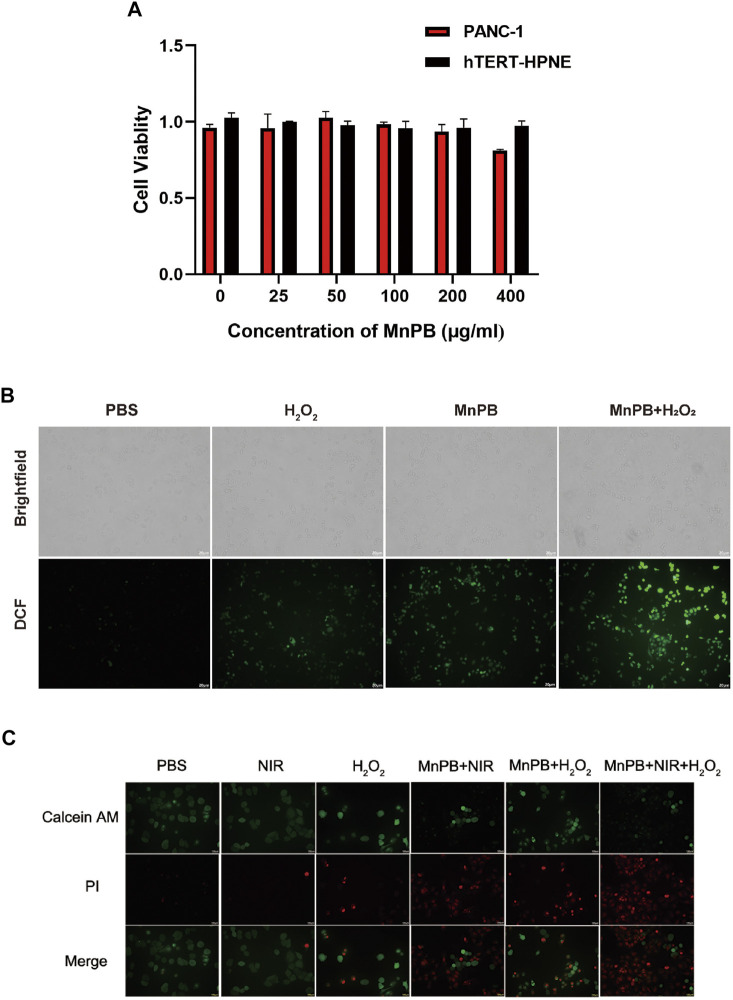
Cell viability and cell death in PC cells with different treatments *in vitro* **(A)** viability of PANC-1 cells and hTERT-HPNE cells with the treatment of MnPB NPs for 24 h. **(B)** The brightfield images and darkfield images of ROS level in PANC-1 cells. **(C)** Images of Calcein-AM and PI co-stained PANC-1 cells in six groups.

We conducted CCK-8 assays to access the biocompatibility and cytotoxicity of MnPB NPs in 2 cell lines including PANC-1 cells and hTERT-HPNE cells. MnPB NPs were incubated with both cell lines with different concentrations (0, 25, 50, 100, 200, 400 μg/ml) for 24 h. As shown in Figure 3A, the results indicated that the viabilities of both cell lines were above 80% even in the high MnPB NPs concentration incubation (400 μg/ml), suggesting MnPB NPs have good biocompatibility and low cytotoxicity. The CDT has attracted lots of attention based on high selectivity in tumors and fewer side effects in normal tissues during the past 5 years (Xu et al., 2021). The production of ROS is a significant part of CDT. To examine whether MnPB NPs could induce ROS, we used DCFH-DA to detect ROS in PANC-1 cells, which could transfer to 2′,7′-dichlorofluorescein (DCF) under the stimulation of intracellular ROS. We observed H_2_O_2_ group and MnPB NPs groups showed weak increases in DCF intensity, while MnPB NPs combined with H_2_O_2_ group showed strong and extensive increases (Figure 3B). Calcine-AM/PI test was used to evaluate the anti-tumor efficiency of MnPB NPs. The PANC-1 cells exhibited extensive green fluorescence, as well as no red fluorescence was observed. Moreover, H_2_O_2_ slightly increased the PI-positive death cells. In contrast, the PANC-1 cells treated with MnPB NPs displayed strong red fluorescence indicating remarkable cell death. There were more PI-positive cells in the group treated with MnPB NPs, NIR and H_2_O_2_ than in the other five groups, which demonstrated the viability of these cells was very low (Figure 3C).

### The MnPB NPs inhibit PC cell migration

**FIGURE 4:**
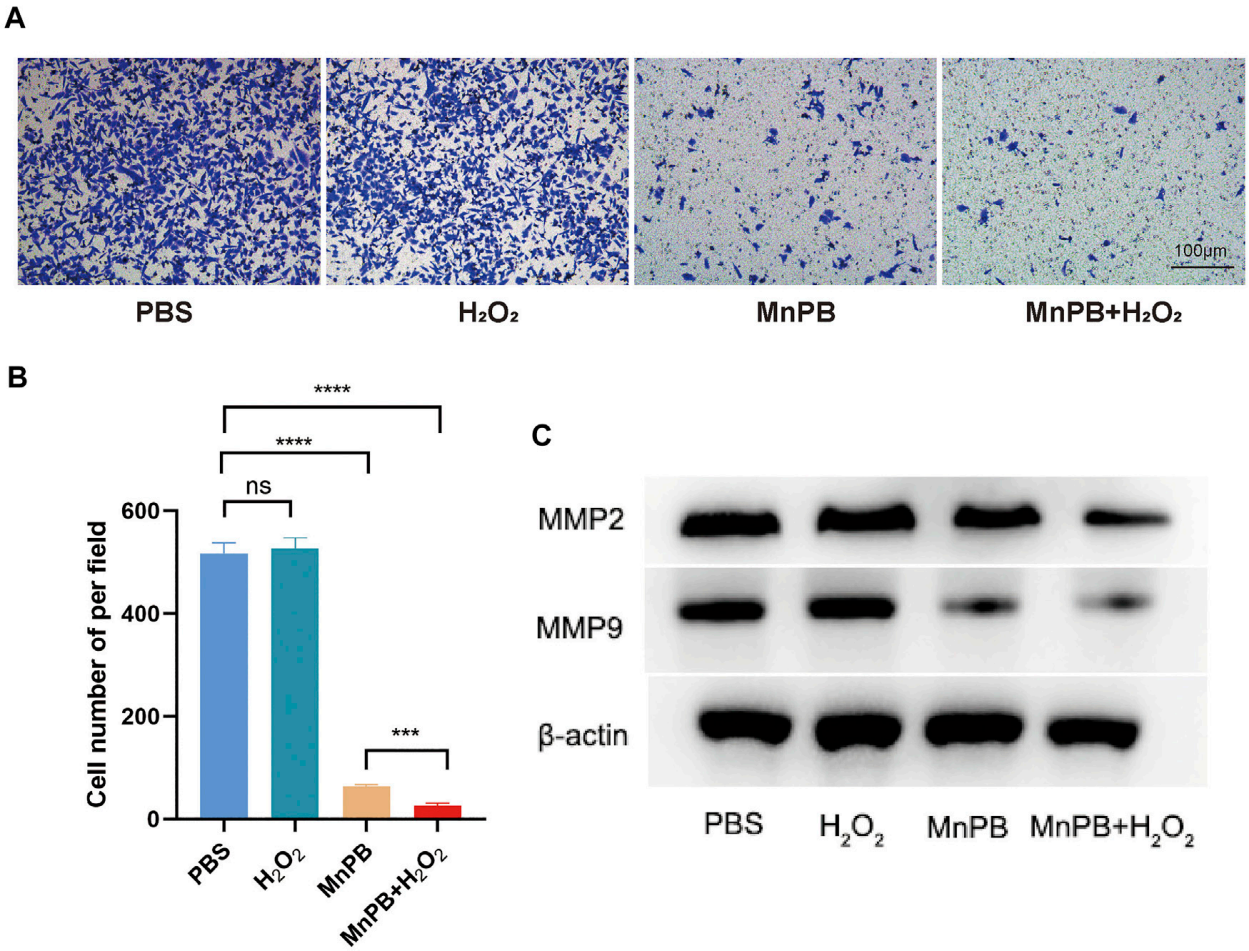
The MnPB NPs inhibit the migration of PANC-1 cells. **(A)** Tanswell assay of PANC-1 cells after MnPB NPs treatment for 48 hours. **(B)** The statistic analysis of transwell migration experiments. **(C)** The expression of MMP2 and MMP9 in four groups. *p < 0.05; **p < 0.01; ***p < 0.001; ****p < 0.0001; ns: no significance.

The process of metastasis, one of the malignant biological behaviors of tumors, involves the transduction of a series of signaling pathways and the expression of key downstream genes. To further assess whether MnPB NPs could influence the mobility of PANC-1 cells, we investigated transwell migration assays. The results certified that MnPB NPs, as well as MnPB NPs + H_2_O_2_, could significantly suppress the migration of PANC-1 cells (Figures 4A,B). What’s more, MMP2 and MMP9 had been reported closed related to migration and invasion of cancers (Zhao et al., 2020; Jiang and Li, 2021; Le et al., 2022). Western blotting was conducted to examine whether the treatment of MnPB NPs could decline the expression some EMT-related proteins. The results demonstrated that MMP2 and MMP9 decreased compared with the control group (Figure 4C). These results were consistent with the study published previously (Fang et al., 2022). Overall, these results indicated that MnPB NPs could inhibit migration of PANC-1 cells.

### The MnPB NPs induce ferroptosis *via* the MAPK pathway

**FIGURE 5:**
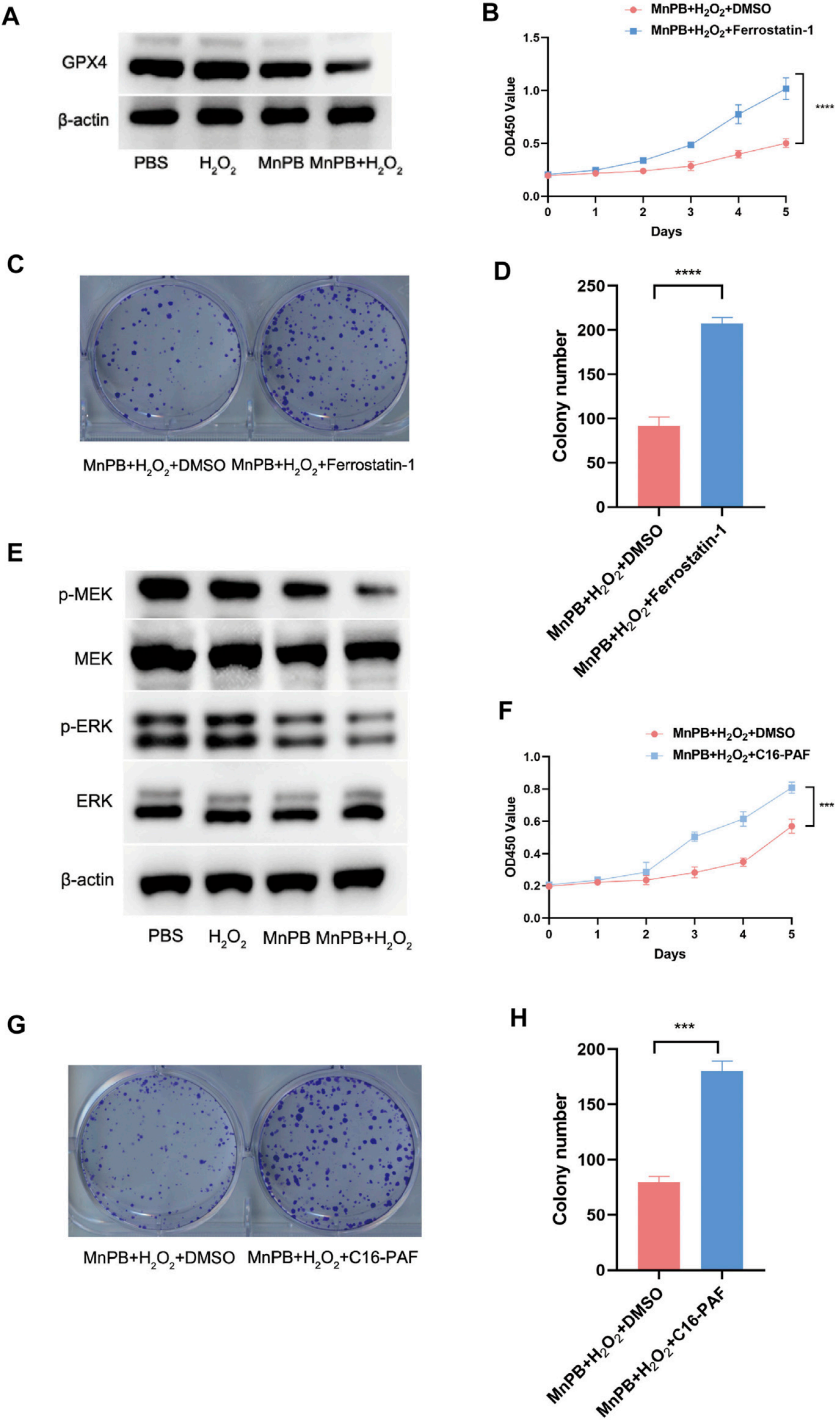
The MnPB NPs promote ferroptosis in PANC-1 cells through the MAPK signal pathway. **(A)** The expression level of GPX4 in different treatments. **(B)** Cell viability was analyzed by CCK-8 assay at the indicated time in PANC-1 cells with DMSO or Ferrostain-1. **(C)** The colony formation assay of PANC-1 cells with DMSO or Ferrostain-1. **(D)** The statistical analysis of colony formation assay of PANC-1 cells with DMSO or Ferrostain-1. **(E)** Western blotting showed the effects of different treatments on the MAPK pathway in PANC-1 cells. **(F)** Cell viability was measured by CCK-8 assay at the indicated time in PANC-1 cells with DMSO or C16-PAF. **(G)** The colony formation assay of PANC-1 cells with DMSO or C16-PAF. **(H)** The statistical analysis of colony formation assay of PANC-1 cells with DMSO or C16-PAF. *p < 0.05; **p < 0.01; ***p < 0.001; ****p < 0.0001.

Multiple previous studies have demonstrated that excess ROS could lead to ferroptosis. We conducted western blotting to confirm whether MnPB NPs could induce ferroptosis in the PC cells. The results indicated that GPX4, the marker of ferroptosis, was downregulated in the group of MnPB NPs + H_2_O_2_ (Figure 5A), suggesting MnPB NPs induced ferroptosis. Moreover, Ferrostatin-1, a kind of ferroptosis inhibitor, was used for the rescue experiment. As shown in Figures 5B–D, Ferrostatin-1 enhanced the proliferation ability inhibited by MnPB NPs + H_2_O_2_. In addition, The MAPK pathway involvement in ferroptosis has been reported in previous studies. Therefore, we explored whether the MAPK pathway was responsible for the ferroptosis in PANC-1 cells induced by MnPB NPs. The results showed p-MEK and p-ERK were decreased in the group of MnPB NPs and MnPB NPs + H_2_O_2_ (Figure 5E), while total MEK 1/2 and ERK 1/2 expression displayed no change. Then we used C16-PAF to activate the MAPK signal pathway. Interestingly, we found the proliferation ability inhibited by MnPB NPs + H_2_O_2_ was markedly reversed by C16-PAF (Figures 5F–H), suggesting MnPB NPs + H_2_O_2_ mediated cell proliferation *via* the MAPK signal pathway. As displayed in Supplemental Figure S1, the expression of GPX4 also increased with the treatment of C16-PAF. Therefore, these data confirmed that MnPB NPs could induce ferroptosis through the MAPK pathway.

### The MnPB NPs inhibit the growth of tumor *in vivo*

**FIGURE 6:**
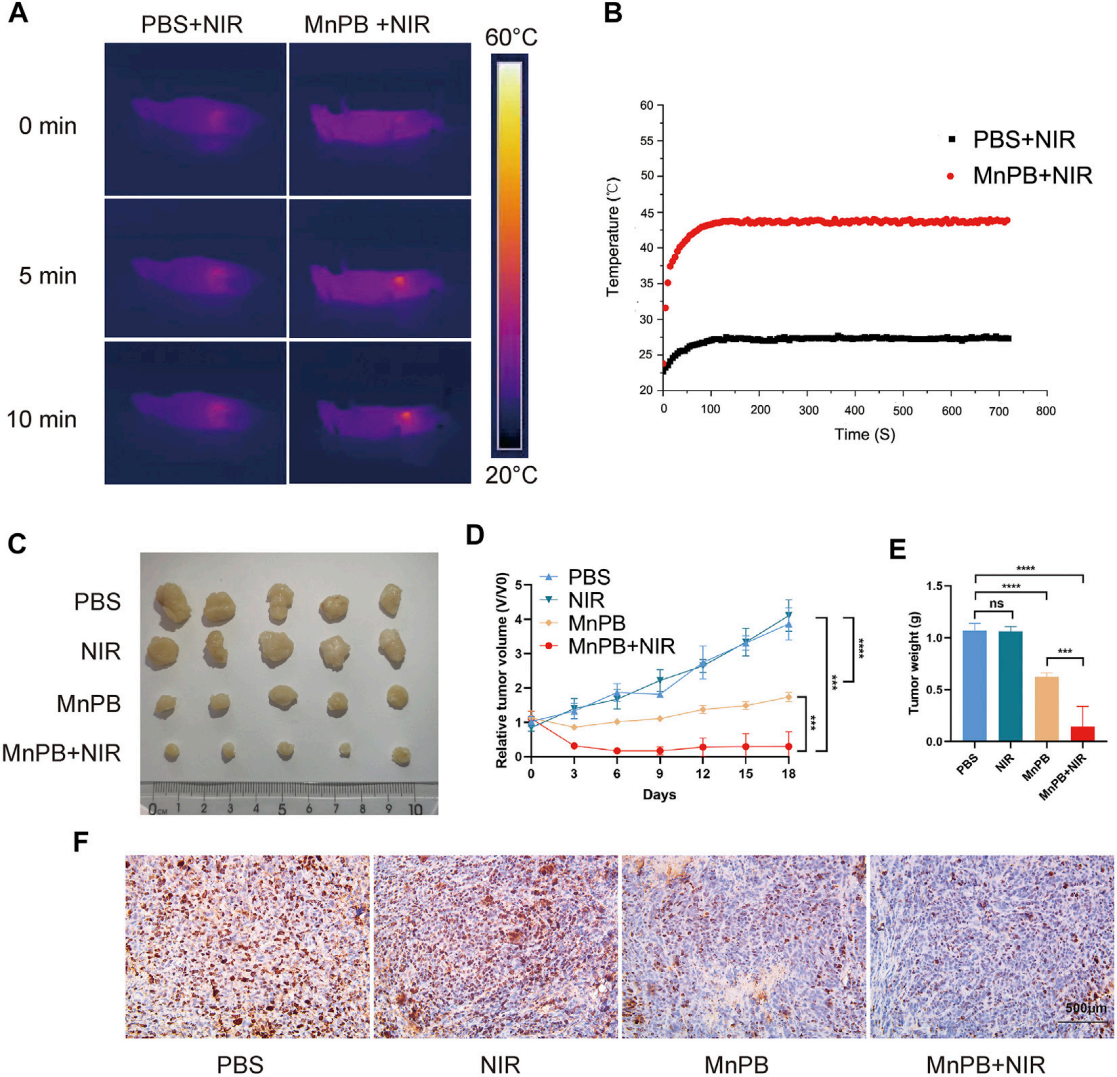
The MnPB NPs inhibit the proliferation of PANC-1 cells *in vivo* **(A)** The infrared thermography pictures of mice under laser irradiation. **(B)** Profile of temperature in the tumor site. **(C)** Digital pictures of each group after varied treatments. **(D)** The tumor volume changes over 18 days. **(E)** Differences between the weight of the subcutaneous tumors with different treatments. **(F)** The immunohistochemical stain images of ki67 in groups with different treatments. *p < 0.05; **p < 0.01; ***p < 0.001; ****p < 0.0001; ns: no significance.

PTT combined with CDT for increasing the tumor treatment performance has aroused heated discussion. To further explore the therapeutic capacity of MnPB NPs on tumor proliferation *in vivo*, we injected 1*10^6^ PANC-1 cells into BALB/c nude mice subcutaneously. After 2 weeks, the volume of tumors reached about 100 mm^3^. For investigating the combination of PTT and CDT to treat tumors synergistically, the mice were randomly divided into four groups (n = 5, per group); 1) PBS, 2) NIR, 3) MnPB NPs, 4) MnPB NPs + NIR. In group 1 and 2, we injected 200 ul PBS into mice *via* tail vein. For group 2, Lasers at 808 nm (1.0 W/cm^2^) were used to irradiate. At the same time, we recorded the infrared thermal image as well as temperature. Mice of group 3 and group 4 were injected with 200 μl MnPB NPs (2 mg/ml) *via* the tail vein. After 12 h, the mice of group 4 were irradiated with an 808 nm NIR laser. The temperature in group 4 rapidly rose to 43.8°C after laser irradiation, which caused irreversible damage, while the temperature of mice in group 2 was less than 28°C (Figures 6A,B). After 18 days, we measured tumor volumes and weights of four groups for a reassessment of the synergistic effect of CDT and PTT. When PTT and CDT are combined in group 4, tumor ablation was significantly increased compared to the other three groups (Figures 6C–E). Next, we applied IHC to evaluate the proliferation status of PC cells, the results indicated that MnPB NPs with NIR could significantly inhibit the growth of PC (Figure 6F).

### *In vivo* biosafety of MnPB NPs

**FIGURE 7:**
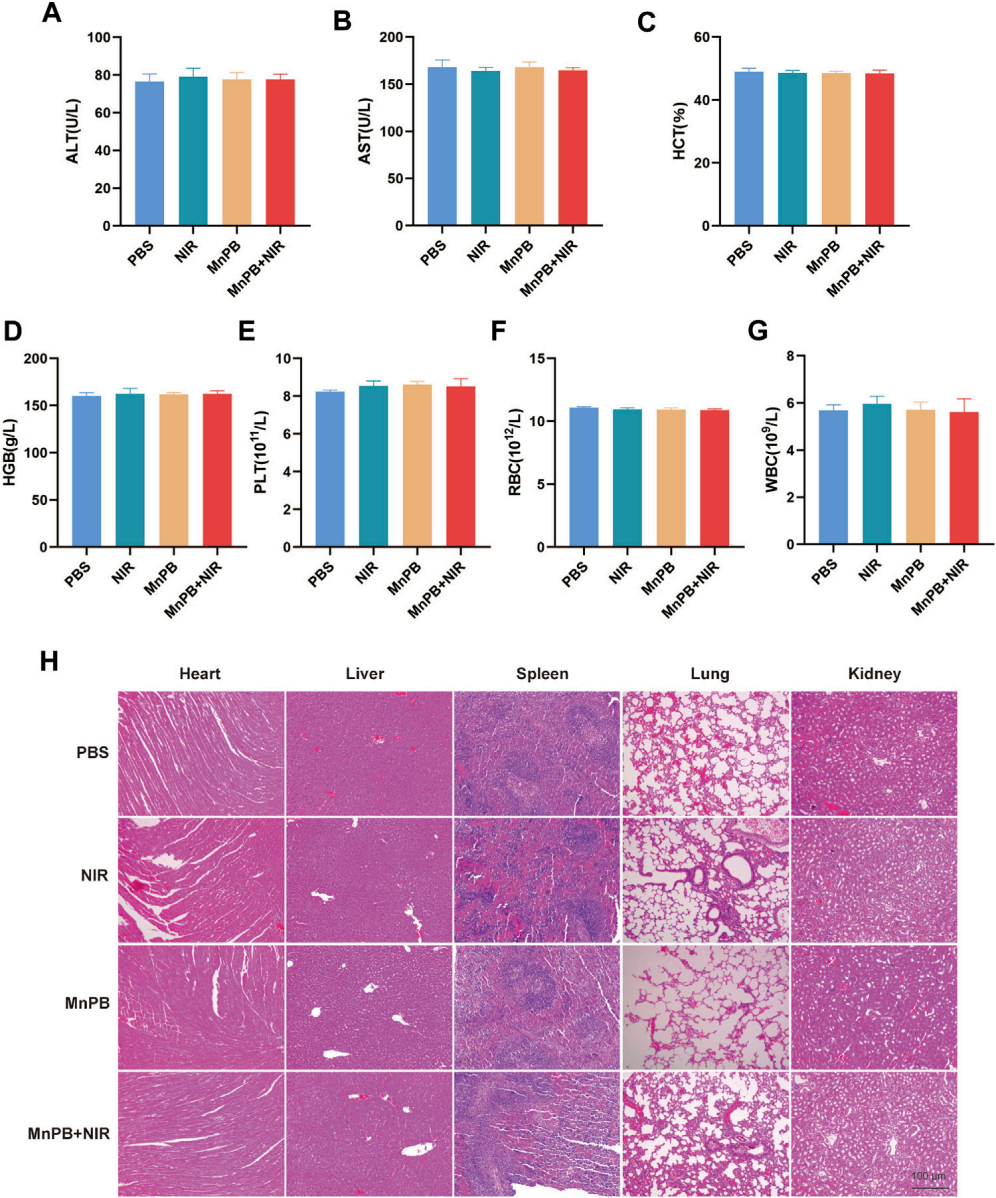
The biosafety of MnPB NPs *in vivo* **(A–G)** the results of liver function and blood tests in different groups. **(H)** HE staining of five major organs in different groups.

Biosafety is very significant for biomaterials application. Therefore, blood routine examination, and liver function test were carried out for blood/liver damage evaluations. The results demonstrated there were few differences among all groups (Figures 7A–G), indicating the treatment of MnPB NPs was safe. Moreover, we conducted HE staining of four groups. The results showed that NIR, MnPB NPs, and MnPB NPs + NIR groups had obscure adverse effects in various organs compared with the PBS group (Figure 7H). In summary, good biosafety of MnPB NPs is guaranteed.

## Discussion

Pancreatic cancer is a well-known deadly disease with similar mortality and morbidity, and its incidence continues to increase, while 5-years relative survival remains the lowest (9%) in all cancers (Chen et al., 2021; Tonini and Zanni, 2021). Furthermore, most pancreatic cancer patients undergo recurrence and metastasis. Despite advances in surgical approaches, its poor prognosis has not improved over the past few decades (Cai et al., 2021). More research is needed to explore and develop new treatments. PB NPs have been widely used in biomedical fields, including staining in histological experiments and targeted drug delivery in cancers, due to their advantages of biocompatibility, affordable production cost, and easy synthesis, which suggests these unique properties make PB NPs potential candidates for a variety of medical applications (Butler and Sadler, 2013; Gautam et al., 2018; Qin et al., 2018). PB NPs exhibit excellent phototherapeutic effects because they have a wide absorption range (500–900 nm) in the near infrared (NIR) region. The FDA has approved PB NPs for the treatment of diseases caused by radiation exposure (Sheikh et al., 2012; Aaseth et al., 2019). Moreover, researchers have identified PB NPs as new photothermal conversion agents for cancer treatment using NIR (Patra, 2016; Haque and Patra, 2021). However, the efficiency of PTT alone is limited because it cannot completely inhibit the tumor. Therefore, combining other metal ions with PB NPs to amplify the advantages of PTT or CDT is expected to become a promising cancer treatment strategy in the future (Wen et al., 2022). In this study, we added an appropriate amount of manganese ions into PB NPs to construct a novel nanoparticle for pancreatic cancer treatment. The keys to PTT are photothermal agents. When photothermal agents are irradiated by the light of an appropriate wavelength, they absorb photon energy and interaction occurred between stimulated molecules and surrounding molecules. This interaction leads to the production of heat, which in turn kills tumor cells. To confirm that MnPB NPs are reliable, several experiments were conducted in this study. Firstly, we evaluated the biosafety of MnPB NPs through the CCK-8 assay. The results showed that MnPB NPs had not much effect on pancreatic cancer cells as well as normal pancreatic cells even if the concentration was up to 400 μg/ml. Moreover, biosafety experiments *in vivo* also confirmed that the MnPB NPs did not cause damage to five major organs. MnPB NPs have a lethal effect on pancreatic cancer cells under near-infrared irradiation. In addition, the results of animal experiments showed the same conclusion, and the adjacent normal tissues were not affected. Briefly, MnPB NPs dissolve manganese ion and activate the Fenton reaction in the TME. Under the catalysis of MnPB NPs, the reaction is as follows:

$$Fe^{2+}+H_{2}O_{2}\to Fe^{3+}+•OH+OH^{-}$$ (1)

$$H_{2}O_{2}+Mn^{2+}\to Mn^{2+}+•OH+OH^{-}$$ (2)

On the one hand, CDT produced excessive •OH, which would oxidize many important cellular components including DNA, proteins, and lipids. Excessive oxidation could lead to irreversible cell death, especially ferroptosis (Li J. et al., 2020). On the other hand, to some extent, the O_2_ catalyzed by MnPB NPs could alleviate tumor hypoxia, which was regarded as the key to drug resistance and tumor metastasis (Harris, 2002; Popper, 2016; Jing et al., 2019). Hence, MnPB NPs are of great significance in the treatment of PC, which could bring Fenton reaction. DCFH-DA fluorescence intensity measurement showed that MnPB NPs significantly upregulated the ROS level of PANC-1 cells under H_2_O_2_ conditions. In addition, we speculated that the cell death caused by MnPB NPs was ferroptosis induced by excessive ROS. Then the change of GPX4, the marker of ferroptosis, was examined by western blotting in four groups. The results indicated ferroptosis in PANC-1 cells was caused by MnPB NPs.

We further explored the mechanism of ferroptosis that occurred in tumor cells. Previous studies revealed that ferroptosis was regulated by some signal pathways. The MAPK pathway plays a crucial role in ferroptosis (Wang et al., 2022c; Ko et al., 2022; Ma et al., 2022). It is reported that the activation of the MAPK pathway is necessary for the ferroptosis of PC cells. Here, western blotting was conducted to confirm the MAPK pathway was suppressed by MnPB NPs. Besides, the phenotype of PANC-1 cells was rescued by C16-PAF, a kind of MAPK pathway activator. Interestingly, the expression of GPX4 was also upregulated by C16-PAF. Therefore, we declared that ferroptosis was regulated by MAPK pathway in PANC-1 cells.

## Conclusion

We synthesized MnPB NPs following a stepwise reaction method. MnPB NPs have satisfactory biocompatibility *in vivo* and *in vitro*. Moreover, MnPB NPs have great potential in synergistic treatments of CDT and PTT, which could produce strong anti-tumor effects, especially inhibiting tumor proliferation and metastasis efficiently. The anti-tumor functions were mediated by the MAPK pathway. Our research indicated the potential of MnPB NPs to develop a new therapeutic strategy for PC. However, more detailed safety and pharmacokinetics studies should be conducted to bring further applications to the clinic.

## Data Availability

The original contributions presented in the study are included in the article/Supplementary Material, further inquiries can be directed to the corresponding authors.
