# Supplementary figures and images for: Manganese-based Prussian blue nanoparticles inhibit tumor proliferation and migration via the MAPK pathway in pancreatic cancer
Source: Front Chem. 2022 Oct 24;10:1026924. doi: 10.3389/fchem.2022.1026924 (PMC9638070; doi:10.3389/fchem.2022.1026924)

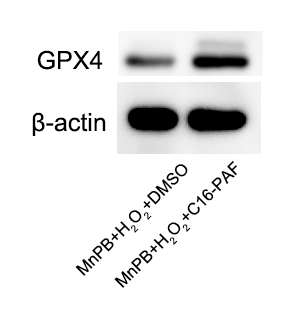

Supplement: Supplementary file 1 [file Image1.PNG]
